# Allergy-related diseases in childhood and risk for abdominal pain-related functional gastrointestinal disorders at 16 years—a birth cohort study

**DOI:** 10.1186/s12916-021-02069-3

**Published:** 2021-09-16

**Authors:** Jessica Sjölund, Inger Kull, Anna Bergström, Jacob Järås, Jonas F. Ludvigsson, Hans Törnblom, Magnus Simrén, Ola Olén

**Affiliations:** 1 Department of Molecular and Clinical Medicine, Institute of Medicine, Sahlgrenska Academy, University of Gothenburg, Box 428, 405 30 Gothenburg, Sweden; 2 Department of Clinical Science and Education, Södersjukhuset, Karolinska Institutet, Stockholm, Sweden; 3 Sachs’ Children’s Hospital, Stockholm, Sweden; 4 Institute of Environmental Medicine, Karolinska Institutet, Stockholm, Sweden; 5 Centre for Occupational and Environmental Medicine, Stockholm County Council, Stockholm, Sweden; 6 Division of Clinical Epidemiology, Department of Medicine Solna, Karolinska Institutet, Stockholm, Sweden; 7 Department of Medical Epidemiology and Biostatistics, Karolinska Institutet, Stockholm, Sweden; 8 Department of Paediatrics, Örebro University Hospital, Örebro, Sweden; 9 Division of Epidemiology and Public Health, School of Medicine, University of Nottingham, Nottingham, UK; 10 Department of Medicine, Columbia University College of Physicians and Surgeons, New York, NY USA; 11 Centre for Functional GI and Motility Disorders, University of North Carolina, Chapel Hill, NC USA

**Keywords:** Allergy, Epidemiology, Functional abdominal pain, Irritable bowel syndrome, Paediatric gastroenterology

## Abstract

**Background:**

Studies on allergy-related diseases in relation to abdominal pain-related functional gastrointestinal disorders (AP-FGIDs) in children are few and results are contradictory. We examined the associations between childhood allergy-related diseases and adolescent AP-FGIDs in general and irritable bowel syndrome (IBS) in particular.

**Method:**

Prospective population-based birth cohort study of 4089 children born in Sweden 1994-1996. We analysed data from 2949 children with complete follow-up at 16 years (y) and no diagnosis of inflammatory bowel disease or coeliac disease at 12y or 16y. Asthma, rhinitis, eczema, and food hypersensitivity (FH) were assessed through questionnaires at 1–2y, 4y, 8y, 12y, and 16y. AP-FGIDs and IBS were assessed through questionnaires at 16y and defined according to the Rome III criteria. Associations between childhood allergy-related diseases and any AP-FGID and IBS and 16y respectively were examined using binomial generalized linear models with a log link function and described as relative risk with 95% confidence intervals.

**Results:**

The prevalence of any AP-FGID and IBS at 16y were 12.0% and 6.0% respectively. Eczema at 1–2y, 4y, and 8y, and FH at 12y and 16y were associated with an increased risk for any AP-FGID at 16y. Asthma and FH at 12y and 16y were associated with an increased risk for IBS at 16y. The relative risk for IBS at 16y increased with increasing number of concurrent allergy-related diseases at 16y, but linear trend for relative risk was only borderline statistically significant (*P* for trend = 0.05).

**Conclusions:**

This prospective population-based study demonstrated positive associations between childhood allergy-related diseases and adolescent AP-FGIDs, including IBS, implicating shared pathophysiology among these disorders.

**Supplementary Information:**

The online version contains supplementary material available at 10.1186/s12916-021-02069-3.

## Background

Childhood abdominal pain-related functional gastrointestinal disorders (AP-FGIDs) are common, affecting 13–25% of children worldwide [1, 2], and have major implications for affected individuals, their families, and society [3]. These disorders are diagnosed using diagnostic criteria, the most recent being the Rome IV criteria, as there are no biological markers or objective clinical findings defining these disorders [4, 5]. One of the most common AP-FGIDs is irritable bowel syndrome (IBS), a disorder characterized by abdominal pain and altered bowel habits [2].

The pathophysiology of AP-FGIDs is not fully understood, but thought to be multifactorial involving complex gut-brain interactions [6]. Proposed pathophysiological mechanisms include low-grade inflammation and immune dysfunction [6], and the relevance of these are supported by clinical observations such as onset of IBS following an episode of infectious gastroenteritis [7], and IBS-like symptoms in a substantial proportion of patients with inflammatory bowel disease (IBD) and coeliac disease (in remission) [8]. Furthermore, both child and adult studies have shown increased infiltration and activation of immune cells such as mast cells and eosinophils in some patients with IBS [9–11] and functional dyspepsia (FD) [12–14].

Allergy-related diseases have been linked to adult AP-FGIDs [15–18]. We have previously shown that allergy-related diseases are positively associated with recurrent functional abdominal pain in pre-adolescents [19]. While several others have explored the associations between asthma [16, 17, 20–25], allergic rhinitis [24, 25], and eczema [24–26] and paediatric AP-FGIDs, many have failed to take temporality into account (cross-sectional studies [20–24]), and of the four studies reporting long-term follow-up [16, 17, 25, 26], none used the Rome III [4] or IV [5] criteria to define AP-FGIDs. Furthermore, it is well known that adults with AP-FGIDs often report postprandial symptom exacerbation [27, 28], and immune-mediated reactions might explain part of this association [29, 30]. Food hypersensitivity (FH), here used as a general term for food-induced symptoms, is commonly reported also in paediatric AP-FGIDs [31–34], but longitudinal population-based studies regarding FH and paediatric AP-FGIDs defined by the Rome criteria are lacking.

Therefore, we aimed to, in a large prospective population-based birth cohort, test the hypothesis that childhood asthma, rhinitis, eczema, and FH are associated with an increased risk for adolescent Rome III-defined AP-FGIDs in general and IBS in particular.

## Methods

### Participants

We collected data from the BAMSE (Swedish abbreviation for *Children*, *Allergy*, *Milieau*, *Stockholm*, *Epidemiology*) study, a prospective population-based birth cohort study of 4089 children born in Sweden from 1994 through 1996 [35]. In BAMSE, parents reported baseline characteristics when their child was 2 months old, allergic symptoms and adverse reactions to food(s) in their child at 1, 2, 4, 8, 12, and 16 years (y), and physician-diagnosed IBD and coeliac disease in their child at 12y and 16y. Children self-reported gastrointestinal symptoms at 16y.

The current study was restricted to children who had answered the questions on gastrointestinal symptoms, at the 16y follow-up. We excluded children with IBD and coeliac disease at 12y and/or 16y. To avoid introducing selection bias, we did not exclude children with a missing report on these diseases, as the proportion of missing reports in the BAMSE cohort exceeds the expected age-specific prevalence of these diagnoses [36].

### Allergy-related diseases

All allergy-related variables were based on parental reports. Detailed age-specific definitions of asthma, rhinitis, eczema, and FH are provided in this article’s additional files (Additional file 1). We assessed both age-specific and overall (any report from 1y through 16y) prevalence. The number of concurrent allergy-related diseases at 16y was used as a proxy for allergy burden at 16y. Missing data on an allergy-related variable was considered a negative report if the parent(s) had participated at the follow-up questionnaire in question.

### AP-FGIDs

At 16y, children answered questions based on the *Questionnaire on Paediatric Gastrointestinal Symptoms-Rome III version* [4] (Additional file 2). Answers were scored according to the paediatric Rome III criteria [4] for IBS, FD, and functional abdominal pain (FAP). Children who fulfilled the criteria for ≥1 of these disorders were classified as having any AP-FGID.

### Statistics

Statistical analyses were performed using STATA Statistical Software (STATA/SE 13.1; StataCorp LP, College Station, TX, USA). All variables were categorical. Prevalence was expressed as percentage of the number of observations available. Baseline characteristics of study participants were compared to the original BAMSE cohort by computing 95% confidence intervals (CI) for study participants and adjusting for finite population sampling [37]. 95% CI not including the prevalence in the original BAMSE cohort were considered statistically significant. Baseline characteristics and prevalence of allergy-related diseases in children with any AP-FGID and IBS respectively were compared to children with no AP-FGID using Pearson´s chi-squared test, and *P* < 0.05 (two-sided) was considered statistically significant.

Age-specific and overall associations between childhood allergy-related diseases and any AP-FGID and IBS at 16y respectively were examined using binomial generalized linear models (GLM) with a log link function and described as relative risk (RR) with 95% CI. Children with no AP-FGID were used as the reference group in all analyses. Models were adjusted for sex, based on its putative role as a risk factor for AP-FGIDs and IBS [1]. All tests were two-sided, 95% CI for RRs not including 1.0 were considered statistically significant, and missing observations were excluded from the analyses. Trend tests of RRs were performed to assess the association between allergy burden at 16y and any AP-FGID and IBS at 16y respectively, and *P* < 0.05 (two-sided) was considered statistically significant.

## Results

### Participants

**Fig. 1:**
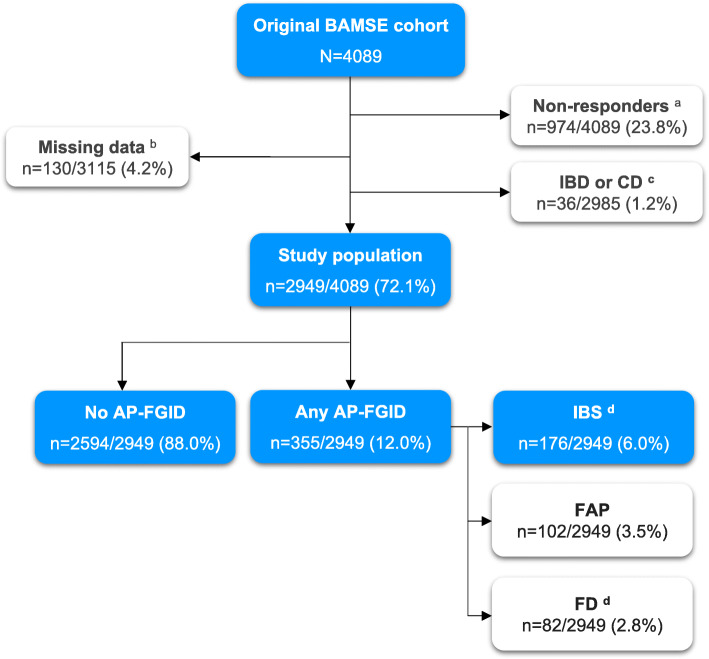
Flow chart. Superscript lowercase letter “a” indicates children who did not participate in the 16y follow-up child questionnaire. Superscript lowercase letter “b” indicates missing data on gastrointestinal symptoms at the 16y follow-up child questionnaire. Superscript lowercase letter “c” indicates at age 12y and/or 16y. Superscript lowercase letter “d” indicates five (*n* = 5) children had overlapping IBS and FD and are included in both the IBS and the FD category in the figure. AP-FGID, abdominal-pain related functional gastrointestinal disorder; CD, coeliac disease; FAP, functional abdominal pain; FD, functional dyspepsia; IBD, inflammatory bowel disease; IBS, irritable bowel syndrome; N, number; y, years

We included 2949 children (Fig. 1). Compared to the original BAMSE cohort, children in the study population were more often the firstborn, exclusively breastfed > 4 months, and of higher socioeconomic status but fewer had a mother who smoked during pregnancy. Absolute differences between study participants and the original BAMSE cohort were however small (Additional file 3).

The prevalence of any AP-FGID and IBS at 16y was 12.0% and 6.0% respectively (Fig. 1). With the exception that any AP-FGID and IBS at 16y were more prevalent in girls, baseline characteristics did not vary with AP-FGID status (Table 1).

**Table 1:** Comparison of baseline characteristics between children with vs. without any AP-FGID and IBS at 16y

	No AP-FGID *n* = 2594	Any AP-FGID *n* = 355		IBS *n* = 176	
	n/n (%)	n/n (%)	***p*** value ^**a**^	n/n (%)	***p*** value ^**a**^
Male sex	1347/2594 (51.9)	115/355 (32.4)	**< 0.001**	64/176 (36.4)	**< 0.001**
Premature birth (< 37 weeks)	146/2594 (5.6)	19/355 (5.4)	0.83	10/176 (5.7)	0.98
Maternal age ≤ 25 years	184/2593 (7.1)	33/355 (9.3)	0.14	18/176 (10.2)	0.12
Low birth weight < 2600 g	123/2568 (4.8)	17/353 (4.8)	0.98	7/174 (4.0)	0.65
Exclusive breastfeeding ≥ 4 months	2041/2538 (80.4)	278/347 (80.1)	0.89	133/170 (78.2)	0.49
Older siblings	1219/2594 (47.0)	165/355 (46.5)	0.86	83/176 (47.2)	0.97
Second hand smoke ^b^	517/2578 (20.1)	75/353 (21.2)	0.60	42/175 (24.0)	0.21
Maternal smoking during pregnancy ^c^	317/2593 (12.2)	42/355 (11.8)	0.83	17/176 (9.7)	0.31
Socioeconomic status of the household					
Blue/lower white-collar worker, other ^d^	780/2584 (30.2)	124/355 (34.9)	0.12	61/176 (34.7)	0.28
Medium white-collar worker ^e^	760/2548 (29.4)	105/355 (29.6)		54/176 (30.7)	
Higher white-collar worker (at least one parent) ^f^	1044/2548 (40.4)	126/355 (35.5)		61/176 (34.7)	
At least one parent has a university/college degree	1453/2572 (56.5)	180/352 (51.1)	0.06	90/174 (51.7)	0.22

^a^Pearson’s chi-squared test. Statistically significant differences (between children with no AP-FGID at 16 years vs. children with any AP-FGID or IBS at 16 years respectively) are shown in bold text

^b^Any parent smoked ≥ 1 cigarette/day at the time of the baseline questionnaire

^c^Mother smoked ≥ 1 cigarette/day during pregnancy

^d^Blue/lower white collar worker include jobs with a normal requirement of ≤ 3 years of education after 9 years of elementary school; other includes students, housewife/man, person on disability pension, and unemployed

^e^Include jobs with a normal requirement of ≥ 3 but ≤ 6 years of education after 9 years of elementary school

^f^Include jobs with a normal requirement of ≥ 6 years of education after 9 years of elementary school

*Abbreviations:* AP-FGID, abdominal pain-related functional gastrointestinal disorder; IBS, irritable bowel syndrome; N, number

### Asthma

**Fig. 2:**
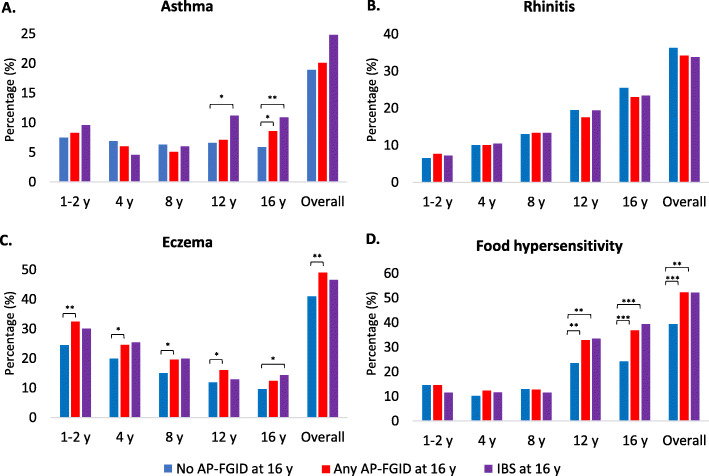
Prevalence of childhood **a** asthma, **b** rhinitis, **c** eczema, and **d** food hypersensitivity. Age-specific and overall (any report from 1y through 16y) prevalence, stratified for children with no AP-FGID, any AP-FGID, and IBS at age 16y respectively. *P < 0.05. **P < 0.01. ***P < 0.001. AP-FGID, abdominal-pain related functional gastrointestinal disorder; IBS, irritable bowel syndrome; y, years

Children with any AP-FGID at 16y more often had concurrent asthma (Fig. 2). In crude GLM, asthma at 16y was positively associated with any AP-FGID at 16y, but this did not remain statistically significant in sex-adjusted models (Additional file 4).

**Fig. 3:**
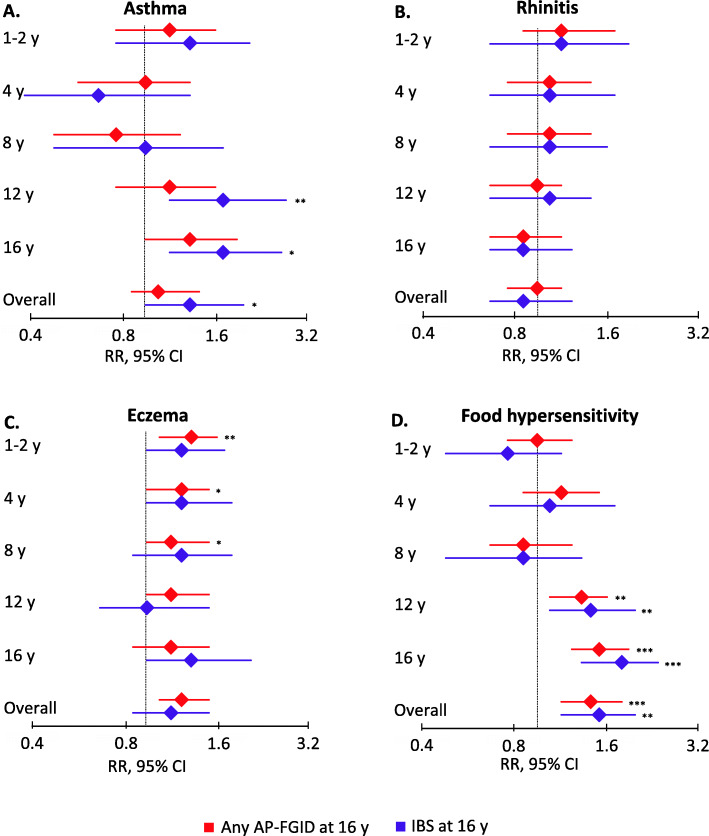
Associations between childhood **a** asthma, **b** rhinitis, **c** eczema, **d** food hypersensitivity, and any AP-FGID and IBS at 16y. Age-specific and overall (any report from 1y through 16y) associations between allergy-related diseases during childhood and any AP-FGID and IBS at 16 years respectively. Associations were assessed in a binomial generalized linear model with a log link function and adjusted for sex. Children with no AP-FGID at age 16y were used as the reference group in all analyses. *P < 0.05. **P < 0.01. ***P < 0.001. AP-FGID, abdominal pain-related functional gastrointestinal disorders; CI, confidence interval; IBS, irritable bowel syndrome; RR, relative risk; y, years

Children with IBS at 16y more often had asthma at 12y and 16y (Fig. 2). In sex-adjusted GLM, overall (any report from 1y through 16y) asthma was positively associated with IBS at 16y (RR 1.4; 95% CI 1.0–2.1), with age-specific associations for asthma at 12y (RR 1.8; 95% CI 1.2–2.9) and 16y (RR 1.8; 95% CI 1.2–2.8) (Fig. 3).

### Rhinitis

The prevalence of rhinitis did not vary with AP-FGID status at 16y (Fig. 2). In crude and sex-adjusted GLM, rhinitis was not associated with any AP-FGID or IBS at 16y (Fig. 3).

### Eczema

Children with any AP-FGID at 16y more often had eczema at 1-2y, 4y, 8y, and 12y (Fig. 2). In sex-adjusted GLM, overall eczema was positively associated with any AP-FGID at 16y (RR 1.3; 95% CI 1.1–1.6), with age-specific associations for eczema at 1–2y (RR 1.4; 95% CI 1.1–1.7), 4y (RR 1.3; 95% CI 1.0–1.6), and 8y (RR 1.2; 95% CI 1.0–1.6) (Fig. 3).

Children with IBS at 16y more often had concurrent eczema (Fig. 2). In sex-adjusted GLM, the RR for IBS at 16y was numerically increased in children with eczema at 1-2y, 4y, and 8y, but this was not statistically significant (Fig. 3). In crude GLM, concurrent eczema was positively associated with IBS at 16y, but this did not remain statistically significant in sex-adjusted models (Additional file 4).

### FH

Children with any AP-FGID at 16y more often had FH at 12y and 16y (Fig. 2). In sex-adjusted GLM, overall FH was positively associated with any AP-FGID at 16y (RR 1.5; 95% CI 1.2–1.9), with age-specific associations for FH at 12y (RR 1.4; 95% CI 1.1–1.7) and 16y (1.6; 95% CI 1.3–2.0) (Fig. 3).

Children with IBS at 16y more often had FH at 12y and 16y (Fig. 2). In sex-adjusted GLM, overall FH was positively associated with IBS at 16y (RR 1.6 95% CI 1.2–2.1), with age-specific associations for FH at 12y (RR 1.5; 95% CI 1.1–2.1) and 16y (RR 1.9; 95% CI 1.4–2.5) (Fig. 3).

### Allergy burden

**Fig. 4:**
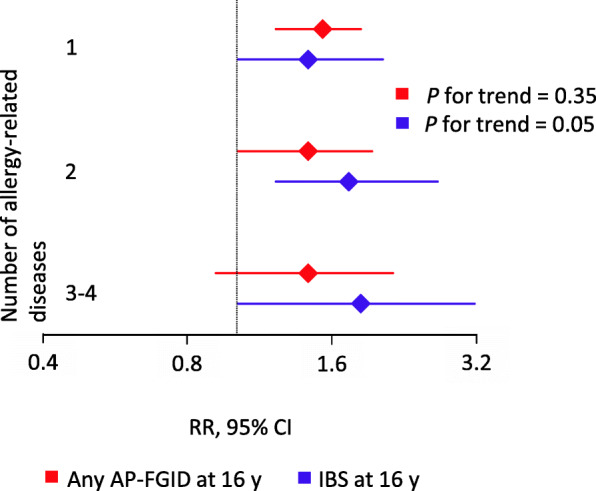
Association between allergy burden at 16y and any AP-FGID and IBS at 16y. The number of concurrent allergy-related diseases (including asthma, rhinitis, eczema, and food hypersensitivity) was used as a proxy for allergy burden at 16y. Associations were assessed in a binomial generalized linear model with a log link function and adjusted for sex. Children with no AP-FGID at age 16 years were used as the reference group in all analyses. AP-FGID, abdominal pain-related functional gastrointestinal disorders; CI, confidence interval; IBS, irritable bowel syndrome; RR, relative risk; y, years

An increasing number of concurrent allergy-related diseases at 16y did not affect the RR for any AP-FGID at 16y (Fig. 4). The RR for IBS at 16y increased with an increasing number of concurrent allergy-related diseases at 16y, but the linear trend for RR was only borderline statistically significant (Fig. 4).

## Discussion

In this large prospective population-based birth cohort study, overall (any report from 1y through 16y) eczema and FH were positively associated with any Rome III-defined AP-FGID at 16y, with age-specific associations for eczema at 1–2y, 4y, and 8y and for FH at 12y and 16y. Further, we found that overall asthma and overall FH were positively associated with Rome III-defined IBS at 16y, with age-specific associations for asthma and FH at 12y and 16y. Targeting immunological mechanisms present in both AP-FGIDs and allergy-related diseases might offer novel therapeutic modalities for AP-FGIDs.

We have previously demonstrated a link between recurrent functional abdominal pain at 12y and concurrent or earlier asthma [19]. When assessing Rome III-defined abdominal pain at 16y, early childhood asthma did not remain associated to AP-FGIDs, whereas asthma at 12y and 16y did. In accordance, the majority of the previous studies including children [16, 17, 20–25] report a high prevalence and/or increased risk for abdominal pain of functional origin [20, 22], AP-FGIDs [21, 23], and IBS [16, 17, 25] when asthma was present. Most studies, however, were cross-sectional [20–24] and of them only Kumari and Colman [21, 23] used the Rome III criteria to define AP-FGIDs (none used the Rome IV criteria). Kumari et al. showed an increased risk for concurrent FD, FAP, and abdominal migraine, but not IBS in asthmatics in a population-based sample of 1101 children in Sri Lanka [21]. This contrasts with our study where asthma was positively associated with IBS, but not AP-FGIDs in general. The discrepancy could be related to differences in sample size, age of assessment (we assessed AP-FGIDs at a later age), and geographical differences of asthma and IBS (we reported a higher prevalence of IBS and lower prevalence of asthma). Colman et al. reported a high prevalence of AP-FGIDs in paediatric patients with persistent asthma and poorer asthma control in patients with AP-FGIDs, but made no attempt to assess the risk for AP-FGIDs in asthmatics relative to non-asthmatics [23]. Of the three longitudinal studies identified, all reported a positive association between asthma and subsequent IBS. Cole and Huerta, however, included both children and adults and made no attempt to report specific paediatric associations [16, 17]. Tan et al. used ICD-codes to identify cases and their results might therefore not be generalizable to the general population [25].

In our previous study in this cohort, we showed positive associations between recurrent functional abdominal and concurrent rhinitis and eczema at 12y [19]. When assessing Rome III-defined abdominal pain at 16y, rhinitis was not associated with AP-FGIDs. We were only able to identify two other studies assessing rhinitis in relation to paediatric AP-FGIDs, with one study reporting an increased risk for subsequent IBS in children with rhinitis [25] and one reporting no association between rhinitis and concurrent IBS [24]. Both studies were however based on selected samples, assessed IBS in younger children, and used outdated or non-accepted criteria to define IBS. The same studies report similar and contradictory associations between eczema and IBS. A third study, report an increased risk for subsequent IBS in children with eczema in Taiwan [26]. While we similarly found positive associations between childhood eczema and adolescent AP-FGIDs in general, we did not find statistically significant associations with adolescent IBS. This discrepancy could be due to the different types of samples (their sample was patient based) and criteria to define IBS (they used ICD-codes under the Rome II era). It is also possible that low statistical power prevented us from detecting statistically significant associations between eczema and IBS.

Compared to healthy peers, adolescents with AP-FGIDs in our study had a higher prevalence of FH at 12y and 16y. This is consistent with previous studies, although our prevalence of FH was lower than what has been reported by others in similar age groups [31–33]. Previous studies, however, were conducted on selected patient samples. Further, they were designed to assess FH and/or dietary interventions, which might have caused selection of children with more food-related symptoms. Longitudinal studies of FH and the risk for subsequent AP-FGIDs are limited. Saps et al. found cow’s milk allergy during the first year of life to predispose for pre-adolescent AP-FGIDs [34], but studies assessing FH in general (as opposed to a single food item) are lacking.

We did not assess allergy-related diseases and discrete AP-FGIDs, but the differences in results for AP-FGIDs vs. IBS suggest that associations may differ between subtypes. Further, although *P* for trend was not statistically significant, the number of concurrent allergy-related diseases increased the RR for IBS, while the RR for any AP-FGID stayed unaffected. This might suggest that allergy is more connected to IBS than to the other AP-FGID subtypes. Except for the study by Kumari et al. discussed above [21], we found no additional studies that have addressed this issue in children. Hence, it would be of clinical and potentially mechanistic interest to stratify for different AP-FGID subtypes when assessing associations to allergy-related diseases in future studies.

Possible explanations of our results include shared pathophysiological mechanisms between asthma, eczema, and FH and adolescent AP-FGIDs. Mast cells and eosinophils, key effector cells in allergy, have been implicated in youth AP-FGIDs. Paediatric IBS has been associated to increased number of mucosal mast cells and mast cells in close proximity to mucosal nerves, which also seem to correlate with pain intensity and frequency [9, 11]. Furthermore, an adult study found that allergic IBS patients had more severe IBS symptoms and higher numbers of mucosal mast cells [38], and others found mucosal immune reactions in IBS patients after mucosal exposure to food antigens [29, 30]. In addition, both mast cells and eosinophils have been implicated in paediatric FD [12, 14]. On the other hand, we did not find any associations between AP-FGIDs and rhinitis, a condition also associated with mast cells and eosinophils. It would be interesting for future studies to investigate mucosal immune activation in individuals with AP-FGIDs and different allergy-related diseases to better understand this overlap.

Further, it is plausible that shared genetic and environmental risk factors may have contributed to our results. Also, allergic children may have increased awareness of bodily symptoms, therefore reporting more symptoms in general. However, at least in adults, the associations between asthma and gastrointestinal symptoms remained when using a control group with other chronic diseases in addition to healthy controls [39]. Furthermore, allergic children report lower quality of life and higher levels of anxiety and depression [40]. It is possible that these effects and not the allergy in itself influence the progress of AP-FGIDs, as anxiety, stress, and symptom awareness are highly implicated internal triggers in the development and/or maintenance of AP-FGIDs [41, 42]. Studies in adults have shown that controlling for mood disorders partly explained the associations between allergy-related diseases and AP-FGIDs [15]. Future studies would need to assess if this also applies to children, as our dataset unfortunately did not allow us to adjust for this.

The most apparent strengths of this study include the population-based design, the large sample size, the evaluation of several allergy-related diseases, the prospective and repeated assessment of allergy-related diseases, and the use of the Rome III criteria [4] to define AP-FGIDs.

As BAMSE was designed to study allergy-related diseases, it is possible that families with allergic children would be more inclined to participate. However, the prevalence of allergy-related diseases during the first years of life was equal in those entering the study and those remaining at 16y, speaking against any major selection bias of more allergic children in our study.

Our definitions of asthma, rhinitis, and eczema have been validated and are highly specific (87–100%) [43, 44]. Further, the prevalence of rhinitis and eczema in our study are in accordance with other population-based studies of similar age groups [45–47]. Although our prevalence of asthma is similar to that reported by some [48], it is lower than that reported by others [46, 49]. While this could be due to actual differences in the populations, we cannot rule out that our strict definition failed to identify all asthma cases.

Our definition of FH did not include a food challenge or objective sensitization measurement (i.e. skin prick test or serum IgE). This is problematic as there is a well-known discrepancy between perceived and confirmed FH in the population [50]. However, also, when using objective measurements to assess FH, there is great diversity in the reported prevalence, highlighting the difficulties with diagnosing FH [50]. In addition, it has been suggested that the immune response related to food antigens in individuals with IBS might not be IgE-driven [29] or limited to local mucosal IgE reactions [30] and thus not detectable with classical food allergy tests such as serum IgE. Furthermore, symptoms in non-classical food allergy may be delayed, which also makes provocation tests problematic [29]. It is likely that the reported FH in our study is caused by a mix of non-immunological and immunological mechanisms. However, until the mechanisms behind FH in AP-FGIDs have been better elucidated, we believe there is relevance in data on perceived FH with regards to the epidemiology of FH in relation to AP-FGIDs.

While we used the Rome III criteria to define AP-FGIDs, case ascertainment did not include a medical evaluation. We did however exclude children with IBD and coeliac disease, and we did previously show that the prevalence of these diagnoses in this cohort are within the expected range [36]. Our dataset prevented us from considering additional organic causes of abdominal pain, but many of these have been shown to be rare in Scandinavian children [51, 52]. Further, we previously reported a clinical follow-up of IBS cases in this cohort, demonstrating a high internal validity of the IBS-classification [36]. Also, the prevalence of AP-FGIDs in our study corresponds well with the reported prevalence of Rome III-defined AP-FGIDs in similar age groups [53, 54]. The reported prevalence of FD increased significantly with the transition from Rome III to Rome IV and the introduction of the diagnostic subgroups *epigastric pain syndrome* and *postprandial distress syndrome (PDS)*, as FD in the case of PDS now can be diagnosed in the absence of abdominal pain [2, 55]. The prevalence of FD and FD-cases experiencing PDS-symptoms in our study would thus likely be higher using the Rome IV criteria. Although we do not report on specific associations between allergies and FD, FD is included in any AP-FGID in our study. Therefore, we cannot rule out that the usage of the Rome IV criteria would affect the associations seen between allergies and any Rome III AP-FGIDs in our study. This might particularly be relevant if the association between allergy and AP-FGIDs is caused by shared pathophysiological mechanisms, as an increase in antral mast cells and eosinophils has been associated with PDS-symptoms such as early satiety but not with epigastric/abdominal pain in children with FD [56, 57].

Unfortunately, we did not know the onset of AP-FGIDs and IBS. Thus, we cannot determine the temporal relationship between allergy-related diseases and AP-FGIDs and IBS in our study. This applies in particular to the associations seen with pre-adolescent asthma and FH, while we find it plausible to assume that eczema likely preceded the development of AP-FGIDs.

## Conclusions

In conclusion, we report that eczema and FH are positively associated with adolescent AP-FGIDs in general, and asthma and FH are positively associated with adolescent IBS. Clinicians faced with these patients should be aware of the co-morbidity between allergy-related diseases and AP-FGIDs. Our results further support the potential role of low-grade inflammation and immune dysregulation in the pathogenesis of paediatric AP-FGIDs.

## Supplementary Information

**Additional file 1.** Age-specific definitions of asthma, rhinitis, eczema, and food hypersensitivity and questionnaire questions used for classification of food hypersensitivity.

**Additional file 2.** Questionnaire questions and answer options in the 16y child-questionnaire used to classify Rome III abdominal pain-related functional gastrointestinal disorders at 16y.

**Additional file 3 **Comparison of baseline characteristics between the entire original BAMSE cohort and study participants. ^a^ Confidence intervals were adjusted for finite population sampling. Statistically significant differences between the entire BAMSE cohort and study participants are shown in bold text. ^b^ Any parent smoked ≥ 1 cigarette/day at the time of the baseline questionnaire. ^c^ Mother smoked ≥ 1 cigarette/day during pregnancy. ^d^ Blue/lower white-collar worker include jobs with a normal requirement of ≤ 3y of education after 9y of elementary school; other includes students, housewife/man, person on disability pension, and un-employed. ^e^ Includes jobs with a normal requirement of ≥ 3y but ≤ 6y of education after 9y of elementary school. ^f^ Includes jobs with a normal requirement of ≥ 6y of education after 9y of elementary school. *Abbreviations:* AP-FGID, abdominal pain-related functional gastrointestinal disorder; CI, confidence interval; N, number; Y, years.

**Additional file 4 **Associations between childhood allergy-related diseases and any AP-FGID and IBS at 16y. ^a^ Cases refers to children with any AP-FGID/IBS at 16y. Non-cases refers to children with no AP-FGID at 16y. The column shows the number of cases and non-cases exposed (yes) vs. unexposed (no) to asthma/rhinitis/eczema/food hypersensitivity at different ages. ^b^ Pearson’s chi-squared test. Statistically significant differences of the AR of developing AP-FGID/IBS at 16y between children exposed and children unexposed to asthma/rhinitis/eczema/food hypersensitivity are shown in bold text. ^c^ Age-specific and overall (any report from 1y through 16y) associations assessed in a binomial generalized linear model with a log link function. Children with no AP-FGID at 16y were used as the reference group in all analyses. Statistically significant associations are marked in bold text. *Abbreviations:* AP-FGID, abdominal pain-related functional gastrointestinal disorder; AR, absolute risk; aRR, adjusted relative risk; CI, confidence interval; IBS, irritable bowel syndrome; N, number; RR, relative risk; Y, years.

## Data Availability

The datasets generated and/or analysed during the current study are not publicly available due to the dataset containing sensitive personal data but are available from the corresponding author on reasonable request and with permission of *Karolinska Institutet*.

## Abbreviations

AP-FGID: Abdominal-pain related functional gastrointestinal disorders
BAMSE: Swedish abbreviation for *Children*, *Allergy*, *Milieau*, *Stockholm*, *Epidemiology*
CI: Confidence interval
FAP: Functional abdominal pain
FD: Functional dyspepsia
FH: Food hypersensitivity
GLM: Generalized linear model
IBD: Inflammatory bowel disease
IBS: Irritable bowel syndrome
RR: Relative risk
Y: Year
