# Supplementary material for: Allergy-related diseases in childhood and risk for abdominal pain-related functional gastrointestinal disorders at 16 years—a birth cohort study
Source: BMC Med. 2021 Sep 16;19:214. doi: 10.1186/s12916-021-02069-3 (PMC8444367; doi:10.1186/s12916-021-02069-3)
Supplement: Supplementary file 1 — Additional file 1. Age-specific definitions of asthma, rhinitis, eczema, and food hypersensitivity and questionnaire questions used for classification of food hypersensitivity. [file 12916_2021_2069_MOESM1_ESM.docx]

| **Additional file 1.** Age-specific definitions of asthma, rhinitis, eczema, and food hypersensitivity and questionnaire questions used for classification of food hypersensitivity. | |
| --- | --- |
| **Asthma** | |
| **At 1-2 years:** ≥3 episodes of wheeze after 3 months (at 1 year) or after 1 year of age (at 2 years) combined either with treatment with inhaled glucocorticosteroids or signs of suspected hyperreactivity (wheezing or severe coughing with exertion  and cold weather, or disturbed coughing at night) without concurrent upper respiratory infection. | |
| **At 4, 8, 12, and 16 years:** ≥4 episodes of wheeze in the last 12 months or ≥1 episode of wheeze during the same time period in combination with occasional or regular treatment with inhaled glucocorticosteroids. | |
| **Rhinitis** | |
| **At 1-2 years:** symptoms from eye or nose after exposure to furred pets or pollen or doctor’s diagnosis of allergic rhinitis from the first 3 months of life (at 1 year) or since the previous questionnaire (at 2 years) | |
| **At 4, and 8 years:** symptoms from eye or nose after exposure to furred pets or pollen or doctor’s diagnosis of allergic rhinitis since the previous questionnaire. | |
| **At 12 years:** symptoms from eye or nose after exposure to furred pets or pollen during the last 12 months or doctor’s diagnosis of allergic rhinitis from the age of 10 years. | |
| **At 16 years:** symptoms from eye or nose after exposure to furred pets or pollen during the last 12 months or doctor’s diagnosis of allergic rhinitis from the age of 12 years. | |
| **Eczema** | |
| **At 1-2 years:** dry skin, itchy rashes for ≥2 weeks at specific location (face or arm  or leg extension surfaces, or arm or leg flexures, or wrist or ankle flexures) of rash or doctor’s diagnosis of eczema after 3 months of age (at 1 year) or 1 year of age (at 2 years). | |
| **At 4 years:** dry skin, itchy rashes for≥2 weeks during the last 12 months at specific location (face or arm or leg extension surfaces, or arm or leg flexures, or wrist or ankle flexures) of rash or doctor’s diagnosis of eczema after 2 years of age. | |
| **At 8 years:** dry skin, itchy rashes for ≥2 weeks during the last 12 months at specific location (face or arm or leg flexures, or wrists or ankles, or neck) of rash or doctor’s diagnosis of eczema after 7 years of age. | |
| **At 12 years:** dry skin, itchy rashes during the last 12 months at specific location (arm or leg flexures, or wrists or ankles, or neck) of rash or doctor’s diagnosis of eczema after 10 years of age. | |
| **At 16 years:** dry skin, itchy rashes during the last 12 months at specific location (arm or leg flexures, or wrists or ankles, or neck) of rash or doctor’s diagnosis of eczema after 12 years of age. | |
| **Food hypersensitivity** | |
| **At 1-2 years:** Reported ≥1 specific symptom to ≥1 specific food item at the time of the questionnaire at 1 and/or 2 years. | |
| **Question questionnaire 1 year:**  Has your child ever had any adverse reaction(s) to food or drink (i.e. vomiting, diarrhoea, eczema, nettle rash, itchiness, swollen lips and/or eyes, runny nose, asthma)? | **Answer options:**   1. No 2. Yes |
| **Question questionnaire 1 year:**  If yes, please specify what reaction(s) your child had? Multiple answers possible. | **Answer options:**   1. Vomiting and/or diarrhoea 2. Eczema 3. Nettle rash 4. Swollen lips and/or eyelids 5. Itchy eyelids and/or runny nose 6. Asthma 7. Other (please specify):______________ |
| **Question questionnaire 1 year:**  Which of the following food item(s) has caused an adverse reaction in your child:   1. Cow’s milk or product containing it (formula, gruel containing milk) 2. Egg 3. Fish 4. Nuts/almonds (excluding peanuts) 5. Peanuts 6. Peas 7. Soy 8. Flour (wheat, rye, barley, oat) 9. Fruit with stones or pips (apple, pear, cherry, plum, peach, nectarine) 10. Other (please specify):______________ | **Answer options:**   1. Has not yet eaten 2. No, does not cause an adverse reaction 3. Yes, cause an adverse reaction |
| **Question questionnaire 2 years:**  Has your child-after the age of one year-ever had any adverse reaction(s) to food or drink (i.e. vomiting, diarrhoea, eczema, nettle rash, itchiness, swollen lips and/or eyes, runny nose, asthma)? | **Answer options:**   1. No 2. Yes |
| **Question questionnaire 2 years:**  If yes, please specify what reaction(s) your child had? Multiple answers possible. | **Answer options:**   1. Vomiting and/or diarrhoea 2. Eczema 3. Nettle rash 4. Swollen lips and/or eyelids 5. Itchy eyelids and/or runny nose 6. Asthma 7. Other (please specify):______________ |
| **Question questionnaire 2 years:**  Which of the following food item(s) has caused an adverse reaction in your child:   1. Cow’s milk or product containing it (formula, gruel containing milk) 2. Egg 3. Fish 4. Nuts/almonds (excluding peanuts) 5. Peanuts 6. Peas 7. Soy 8. Flour (wheat, rye, barley, oat) 9. Fruit with stones or pips (apple, pear, cherry, plum, peach, nectarine) 10. Other (please specify):______________ | **Answer options:**   1. Has not yet eaten 2. No, does not cause an adverse reaction 3. Yes, cause an adverse reaction |
| **At 4 years:** Reported ≥1 specific symptom to ≥1 specific food item after the age of 2 years. | |
| **Question questionnaire 4 years:**  Has your child-after the age of 2 years- ever had any adverse reaction(s) to food or drink? | **Answer options:**   1. No 2. Yes |
| **Question questionnaire 4 years:**  If yes, please specify what reaction(s) your child had? Multiple answers possible. | **Answer options:**   1. Vomiting and/or diarrhoea 2. Eczema 3. Nettle rash 4. Swollen lips or eyelids 5. Itchy eyelids or runny nose 6. Asthma 7. Other (please specify): _____________ |
| **Question questionnaire 4 years:**  Which of the following food item(s) has caused an adverse reaction in your child, after the age of two years:   1. Cow’s milk or product containing it (formula, gruel containing milk) 2. Egg 3. Fish 4. Nuts/almonds (excluding peanuts) 5. Peanuts 6. Peas 7. Soy 8. Flour (wheat, rye, barley, oat) 9. Fruit with stones or pips (apple, pear, cherry, plum, peach, nectarine) 10. Citrus fruit 11. Chocolate 12. Banana | **Answer options:**   1. Has not yet eaten 2. No, does not cause an adverse reaction 3. Yes, cause an adverse reaction 4. Excluded from diet due to an adverse reaction |
| **At 8 years:** Reported ≥1 specific symptom to ≥1 specific food item at the time of the questionnaire at 8 years. | |
| **Question questionnaire 8 years:**  Is your child allergic to any food item? | **Answer options:**   1. No 2. Yes |
| **Question questionnaire 8 years:**  If yes, please specify which reaction(s) your child had to the following:   1. Milk 2. Egg 3. Fish 4. Shellfish 5. Wheat flour 6. Soy 7. Apple 8. Peach 9. Kiwi 10. Avocado 11. Banana 12. Raw carrots 13. Peanuts 14. Nuts/almonds (except peanuts) | **Answer options:**   1. Nose/Eye problems 2. Itching in mouth 3. Trouble breathing 4. Vomiting or diarrhoea 5. Eczema 6. Nettle rash 7. Avoided food item because of previous adverse reaction |
| **At 12 years:** Reported ≥1 specific symptom to ≥1 specific food item during the last 12 months. | |
| **Question questionnaire 12 years:**  Has your child had an adverse reaction to any of the following food items?   1. Milk 2. Egg 3. Fish 4. Shellfish 5. Wheat 6. Soy 7. Sesame 8. Apple, pear, peach, cherry 9. Kiwi 10. Peanut 11. Hazelnut 12. Almond 13. Walnut 14. Cashew 15. Brazil nut 16. Pistachio 17. Other (please specify): _____________ | **Answer options:**   1. No 2. Don’t know/has never eaten 3. Avoids due to previous adverse reaction 4. Yes, has had an adverse reaction in the past but not any longer 5. Yes, has had an adverse reaction during the past 12 months |
| **Question questionnaire 12 years:**  If yes, how did your child react to the following food items? Multiple answers possible.   1. Milk 2. Egg 3. Fish 4. Shellfish 5. Wheat 6. Soy 7. Sesame 8. Apple, pear, peach, cherry 9. Kiwi 10. Peanut 11. Hazelnut 12. Almond 13. Walnut 14. Cashew 15. Brazil nut 16. Pistachio 17. Other (please specify): ______________ | **Answer options:**   1. Asthma or allergy 2. Itchy nose, stuffy nose, runny nose 3. Itchy, red eyes 4. Eczema 5. Nettle rash 6. Vomiting 7. Diarrhoea 8. Severe stomach cramp 9. Swollen face, eyelids, lips 10. Cough or hoarseness 11. Feeling of swollen pharynx or throat 12. Itchiness in mouth, pharynx, ears 13. Pronounced fatigue, decreased awareness 14. Other (please specify): _____________ |
| **At 16 years:** Reported ≥1 specific symptom to ≥1 specific food item during the last 12 months. | |
| **Question questionnaire 16 years:**  Has your child ever reacted to any of the following food items?   1. Milk 2. Egg 3. Fish 4. Shellfish 5. Wheat and other grains 6. Soy 7. Sesame 8. Apple, pear 9. Peach, nectarine, plum, cherry 10. Kiwi 11. Banana 12. Raw carrot 13. Peanut 14. Hazelnut 15. Almond 16. Walnut, pecan 17. Cashew nut, pistachio 18. Brazil nut 19. Other (please specify): _____________ | **Answer options:**   1. No 2. Yes, has reacted during the past 12 months 3. Yes, has reacted before but tolerate it now 4. Avoids because of previous reactions or confirmed allergy test |
| **Question questionnaire 16 years:**  If yes, how did your child react to the following food items? Multiple answers possible.   1. Milk 2. Egg 3. Fish 4. Shellfish 5. Wheat and other grains 6. Soy 7. Sesame 8. Apple, pear 9. Peach, nectarine, plum, cherry 10. Kiwi 11. Banana 12. Raw carrot 13. Peanut 14. Hazelnut 15. Almond 16. Walnut, pecan 17. Cashew nut, pistachio 18. Brazil nut 19. Other (please specify): _____________ | **Answer options:**   1. Breathing difficulties, asthma, cough 2. Itchy nose, stuffy nose, runny nose, itchy eyes 3. Nettle rash 4. Vomiting, stomach pain 5. Swollen face, eyelids, lips 6. Hoarseness 7. Feeling of swollen mouth, pharynx, throat 8. Itchiness in mouth, pharynx, ears 9. Pronounced fatigue, decreased awareness 10. Unconsciousness 11. Other (please specify): _____________ |
