# Supplementary material for: Allergy-related diseases in childhood and risk for abdominal pain-related functional gastrointestinal disorders at 16 years—a birth cohort study
Source: BMC Med. 2021 Sep 16;19:214. doi: 10.1186/s12916-021-02069-3 (PMC8444367; doi:10.1186/s12916-021-02069-3)
Supplement: Supplementary file 2 — Additional file 2. Questionnaire questions and answer options in the 16y child-questionnaire used to classify Rome III abdominal pain-related functional gastrointestinal disorders at 16y. [file 12916_2021_2069_MOESM2_ESM.docx]

| Additional file 2**.** Questions and answer options in the 16y child-questionnaire used to classify Rome III abdominal pain-related functional gastrointestinal disorders at 16y. | |
| --- | --- |
| How often in the past 2 months have you felt abdominal pain or discomfort? | 1. Never or less than once a week 2. 1 time per week 3. Several times per week or more often |
| How often in the past 2 months have you felt pain or discomfort in the area ***above*** your navel? | 1. Never or less than once a week 2. 1 time per week 3. Several times per week or more often |
| How long have you felt pain or discomfort in the area ***above*** your navel? | 1. 1 month or less 2. 2-12 months 3. More than a year |
| In the past 2 months when you have felt pain or discomfort ***above*** your navel how often:  *Did the pain or discomfort decrease after you had a bowel movement?* | 1. Never or on a few occasions (0-25% of the time) 2. Sometimes (50% of the time) 3. Usually or always (75-100% of the time) |
| In the past 2 months when you have felt pain or discomfort ***above*** your navel, how often:  *Was you stool softer or harder than usual?* | 1. Never or on a few occasions (0-25% of the time) 2. Sometimes (50% of the time) 3. Usually or always (75-100% of the time) |
| In the past 2 months when you have felt pain or discomfort ***above*** your navel, how often:  *Did you have bowel movements more or less times than usual?* | 1. Never or on a few occasions (0-25% of the time) 2. Sometimes (50% of the time) 3. Usually or always (75-100% of the time) |
| How often in the past 2 months have you felt pain or discomfort in the area ***around or below*** your navel? | 1. Never or less than once a week 2. 1 time per week 3. Several times per week or more often |
| How long have you felt pain or discomfort in the area ***around or below*** your navel? | 1. 1 month or less 2. 2-12 months 3. More than a year |
| In the past 2 months when you have felt pain or discomfort ***around or below*** your navel, how often:  *Did the pain or discomfort decrease after you had a bowel movement?* | 1. Never or on a few occasions (0-25% of the time) 2. Sometimes (50% of the time) 3. Usually or always (75-100% of the time) |
| In the past 2 months when you have felt pain or discomfort ***around or below*** your navel, how often:  *Was you stool softer or harder than usual?* | 1. Never or on a few occasions (0-25% of the time) 2. Sometimes (50% of the time) 3. Usually or always (75-100% of the time) |
| In the past 2 months when you have felt pain or discomfort ***around or below*** your navel, how often:  *Did you have bowel movements more or less times than usual?* | 1. Never or on a few occasions (0-25% of the time) 2. Sometimes (50% of the time) 3. Usually or always (75-100% of the time) |
