# Supplementary material for: Allergy-related diseases in childhood and risk for abdominal pain-related functional gastrointestinal disorders at 16 years—a birth cohort study
Source: BMC Med. 2021 Sep 16;19:214. doi: 10.1186/s12916-021-02069-3 (PMC8444367; doi:10.1186/s12916-021-02069-3)
Supplement: Supplementary file 3 — Additional file 3 Comparison of baseline characteristics between the entire original BAMSE cohort and study participants. a Confidence intervals were adjusted for finite population sampling. Statistically significant differences between the entire BAMSE cohort and study participants are shown in bold text. b Any parent smoked ≥ 1 cigarette/day at the time of the baseline questionnaire. c Mother smoked ≥ 1 cigarette/day during pregnancy. d Blue/lower white-collar worker include jobs with a normal requirement of ≤ 3y of education after 9y of elementary school; other includes students, housewife/man, person on disability pension, and un-employed. e Includes jobs with a normal requirement of ≥ 3y but ≤ 6y of education after 9y of elementary school. f Includes jobs with a normal requirement of ≥ 6y of education after 9y of elementary school. Abbreviations: AP-FGID, abdominal pain-related functional gastrointestinal disorder; CI, confidence interval; N, number; Y, years. [file 12916_2021_2069_MOESM3_ESM.docx]

| **Additional File 3.** Comparison of baseline characteristics between the entire original BAMSE cohort and study participants | | | | |
| --- | --- | --- | --- | --- |
|  | **Original BAMSE cohort**  **N=4089** | | **Study participants**  **n=2949** | |
|  | **n/N** | **%** | **n/n** | **%**  **(95% CI)^a^** |
| Male sex | 2065/4089 | 50.5 | 1462/2949 | 49.6 (48.6-50.5) |
| Premature birth (<37 weeks) | 227/4089 | 5.6 | 165/2949 | 5.6 (5.2-6.0) |
| Maternal age ≤25 years | 319/4088 | 7.8 | 217/2948 | 7.4 (6.9-7.9) |
| Birth weight <2600g | 189/4044 | 4.7 | 140/2921 | 4.8 (4.4-5.2) |
| Exclusive breastfeeding ≥ 4 months | 3116/3919 | 79.5 | 2319/2885 | **80.4 (79.6-81.1)** |
| Older siblings | 1980/4088 | 48.4 | 1384/2949 | **46.9 (46.0-47.9)** |
| Second-hand tobacco smoke ^b^ | 855/4067 | 21.0 | 592/2931 | 20.2 (19.4-21.0) |
| Maternal smoking during pregnancy ^c^ | 531/4088 | 13.0 | 359/2948 | **12.2 (11.6-12.8)** |
| Socioeconomic status of the household |  |  |  |  |
| Blue/lower white collar worker, other ^d^ | 1354/4072 | 33.3 | 904/2939 | **30.8 (29.9-31.6)** |
| Medium white collar worker ^e^ | 1179/4072 | 29.0 | 865/2939 | 29.4 (28.6-30.3) |
| Higher white collar worker (at least one parent) ^f^ | 1539/4072 | 37.8 | 1170/2939 | **39.8 (38.9-40.7)** |
| At least one parent has a university/college degree | 2161/4046 | 53.4 | 1633/2924 | **55.9 (54.9-56.8)** |
| ^a^ Confidence intervals were adjusted for finite population sampling. Statistically significant differences between the entire BAMSE cohort and study participants are shown in bold text.  ^b^ Any parent smoked ≥ 1 cigarette/day at the time of the baseline questionnaire.  ^c^ Mother smoked ≥ 1 cigarette/day during pregnancy.  ^d^ Blue/lower white-collar worker include jobs with a normal requirement of **≤** 3y of education after 9y of elementary school; other includes students, housewife/man, person on disability pension, and un-employed.  ^e^ Includes jobs with a normal requirement of ≥ 3y but **≤** 6y of education after 9y of elementary school.  ^f^ Includes jobs with a normal requirement of ≥ 6y of education after 9y of elementary school.  *Abbreviations:* AP-FGID, abdominal pain-related functional gastrointestinal disorder; CI, confidence interval; N, number; Y, years. | | | | |
