# Supplementary material for: Allergy-related diseases in childhood and risk for abdominal pain-related functional gastrointestinal disorders at 16 years—a birth cohort study
Source: BMC Med. 2021 Sep 16;19:214. doi: 10.1186/s12916-021-02069-3 (PMC8444367; doi:10.1186/s12916-021-02069-3)
Supplement: Supplementary file 4 — Additional file 4 Associations between childhood allergy-related diseases and any AP-FGID and IBS at 16y. a Cases refers to children with any AP-FGID/IBS at 16y. Non-cases refers to children with no AP-FGID at 16y. The column shows the number of cases and non-cases exposed (yes) vs. unexposed (no) to asthma/rhinitis/eczema/food hypersensitivity at different ages. b Pearson’s chi-squared test. Statistically significant differences of the AR of developing AP-FGID/IBS at 16y between children exposed and children unexposed to asthma/rhinitis/eczema/food hypersensitivity are shown in bold text. c Age-specific and overall (any report from 1y through 16y) associations assessed in a binomial generalized linear model with a log link function. Children with no AP-FGID at 16y were used as the reference group in all analyses. Statistically significant associations are marked in bold text. Abbreviations: AP-FGID, abdominal pain-related functional gastrointestinal disorder; AR, absolute risk; aRR, adjusted relative risk; CI, confidence interval; IBS, irritable bowel syndrome; N, number; RR, relative risk; Y, years. [file 12916_2021_2069_MOESM4_ESM.docx]

| Additional file 4. Associations between childhood allergy-related diseases and any AP-FGID and IBS at 16y. | | | | | | | | | | | | | | | | | | | |
| --- | --- | --- | --- | --- | --- | --- | --- | --- | --- | --- | --- | --- | --- | --- | --- | --- | --- | --- | --- |
|  | | **Outcome: Any AP-FGID** | | | | | | | | | **Outcome: IBS** | | | | | | | | |
|  |  |  | | | **Crude** | | | **Adjusted for sex** | | |  | | | **Crude** | | | **Adjusted for sex** | | |
|  |  | **n cases/non-cases ^a^** | **AR (%)** | ***P ^b^*** | **RR ^c^** | **95% CI** | ***P*** | **aRR ^c^** | **95% CI** | ***P*** | **n cases/non-cases ^a^** | **AR (%)** | ***P* ^b^** | **RR ^c^** | **95% CI** | ***P*** | **aRR ^c^** | **95% CI** | ***P*** |
| **Asthma** | | | | | | | | | | | | | | | | | | | |
| 1-2 years | Yes | 28/187 | 13.0 | 0.59 | 1.1 | 0.8-1.6 | 0.58 | 1.2 | 0.8-1.7 | 0.37 | 16/187 | 7.9 | 0.31 | 1.3 | 0.8-2.1 | 0.31 | 1.4 | 0.8-2.2 | 0.22 |
|  | No | 308/2308 | 11.8 |  |  |  |  |  |  |  | 150/2308 | 6.1 |  |  |  |  |  |  |  |
| 4 years | Yes | 21/175 | 10.7 | 0.54 | 0.9 | 0.6-1.3 | 0.54 | 1.0 | 0.6-1.4 | 0.81 | 8/175 | 4.4 | 0.25 | 0.7 | 0.3-1.3 | 0.26 | 0.7 | 0.4-1.4 | 0.34 |
|  | No | 328/2358 | 12.2 |  |  |  |  |  |  |  | 165/2358 | 6.5 |  |  |  |  |  |  |  |
| 8 years | Yes | 17/157 | 9.8 | 0.40 | 0.8 | 0.5-1.3 | 0.40 | 0.8 | 0.5-1.3 | 0.49 | 10/157 | 6.0 | 0.86 | 1.0 | 0.5-1.8 | 0.86 | 1.0 | 0.5-1.8 | 0.94 |
|  | No | 314/2323 | 11.9 |  |  |  |  |  |  |  | 156/2323 | 6.3 |  |  |  |  |  |  |  |
| 12 years | Yes | 24/164 | 12.8 | 0.74 | 1.1 | 0.7-1.6 | 0.74 | 1.2 | 0.8-1.7 | 0.38 | 19/164 | 10.4 | **0.02** | 1.7 | 1.1-2.7 | **0.02** | 1.8 | 1.2-2.9 | **<0.01** |
|  | No | 313/2308 | 11.9 |  |  |  |  |  |  |  | 151/2308 | 6.1 |  |  |  |  |  |  |  |
| 16 years | Yes | 30/150 | 16.7 | **0.049** | 1.4 | 1.0-2.0 | **0.04** | 1.4 | 1.0-2.0 | 0.06 | 19/150 | 11.2 | **<0.01** | 1.8 | 1.2-2.9 | **<0.01** | 1.8 | 1.2-2.8 | **0.01** |
|  | No | 318/2393 | 11.7 |  |  |  |  |  |  |  | 156/2393 | 6.1 |  |  |  |  |  |  |  |
| Overall | Yes | 61/433 | 12.3 | 0.63 | 1.1 | 0.8-1.4 | 0.63 | 1.1 | 0.9-1.5 | 0.37 | 39/433 | 8.3 | 0.07 | 1.4 | 1.0-2.0 | 0.07 | 1.4 | 1.0-2.1 | **0.04** |
|  | No | 243/1855 | 11.6 |  |  |  |  |  |  |  | 118/1855 | 6.0 |  |  |  |  |  |  |  |
| **Rhinitis** | | | | | | | | | | | | | | | | | | | |
| 1-2 years | Yes | 26/163 | 13.8 | 0.41 | 1.2 | 0.8-1.7 | 0.40 | 1.2 | 0.9-1.8 | 0.25 | 12/163 | 6.9 | 0.73 | 1.1 | 0.6-2.0 | 0.73 | 1.2 | 0.7-2.0 | 0.60 |
|  | No | 310/2332 | 11.7 |  |  |  |  |  |  |  | 154/2332 | 6.2 |  |  |  |  |  |  |  |
| 4 years | Yes | 35/253 | 12.2 | 0.98 | 1.0 | 0.7-1.4 | 0.98 | 1.1 | 0.8-1.5 | 0.69 | 18/253 | 6.6 | 0.86 | 1.0 | 0.7-1.7 | 0.86 | 1.1 | 0.7-1.8 | 0.69 |
|  | No | 314/2280 | 12.1 |  |  |  |  |  |  |  | 155/2280 | 6.3 |  |  |  |  |  |  |  |
| 8 years | Yes | 44/322 | 12.0 | 0.88 | 1.0 | 0.8-1.4 | 0.88 | 1.1 | 0.8-1.5 | 0.61 | 22/322 | 6.4 | 0.92 | 1.0 | 0.7-1.6 | 0.92 | 1.1 | 0.7-1.7 | 0.75 |
|  | No | 287/2158 | 11.7 |  |  |  |  |  |  |  | 144/2158 | 6.3 |  |  |  |  |  |  |  |
| 12 years | Yes | 59/481 | 10.9 | 0.39 | 0.9 | 0.7-1.2 | 0.40 | 1.0 | 0.7-1.2 | 0.73 | 33/481 | 6.4 | 0.99 | 1.0 | 0.7-1.4 | 0.99 | 1.1 | 0.7-1.5 | 0.77 |
|  | No | 278/1991 | 12.3 |  |  |  |  |  |  |  | 137/1991 | 6.4 |  |  |  |  |  |  |  |
| 16 years | Yes | 80/648 | 11.0 | 0.31 | 0.9 | 0.7-1.1 | 0.32 | 0.9 | 0.7-1.2 | 0.52 | 41/648 | 6.0 | 0.55 | 0.9 | 0.6-1.3 | 0.55 | 0.9 | 0.7-1.3 | 0.69 |
|  | No | 268/1895 | 12.4 |  |  |  |  |  |  |  | 134/1895 | 6.6 |  |  |  |  |  |  |  |
| Overall | Yes | 104/831 | 11.1 | 0.47 | 0.9 | 0.7-1.2 | 0.47 | 1.0 | 0.8-1.2 | 0.86 | 53/831 | 6.0 | 0.52 | 0.9 | 0.7-1.2 | 0.52 | 0.9 | 0.7-1.3 | 0.75 |
|  | No | 200/1457 | 12.1 |  |  |  |  |  |  |  | 104/1457 | 6.6 |  |  |  |  |  |  |  |
| **Eczema** | | | | | | | | | | | | | | | | | | | |
| 1-2 years | Yes | 109/611 | 15.1 | **<0.01** | 1.4 | 1.1-1.7 | **<0.01** | 1.4 | 1.1-1.7 | **<0.01** | 50/611  116/1884 | 7.6  5.8 | 0.10 | 1.3 | 0.9-1.8 | 0.10 | 1.3 | 1.0-1.8 | 0.09 |
|  | No | 227/1884 | 10.8 |  |  |  |  |  |  |  |  |  |  |  |  |  |  |  |  |
| 4 years | Yes | 86/504 | 14.6 | **0.04** | 1.3 | 1.0-1.6 | **0.04** | 1.3 | 1.0-1.6 | **0.04** | 44/504 | 8.0 | 0.08 | 1.3 | 1.0-1.9 | 0.08 | 1.3 | 1.0-1.9 | 0.08 |
|  | No | 263/2029 | 11.5 |  |  |  |  |  |  |  | 129/2029 | 6.0 |  |  |  |  |  |  |  |
| 8 years | Yes | 65/371 | 14.9 | **0.03** | 1.3 | 1.0-1.8 | **0.03** | 1.2 | 1.0-1.6 | **0.047** | 33/371 | 8.2 | 0.09 | 1.4 | 1.0-2.0 | 0.09 | 1.3 | 0.9-1.9 | 0.12 |
|  | No | 266/2109 | 11.2 |  |  |  |  |  |  |  | 133/2109 | 5.9 |  |  |  |  |  |  |  |
| 12 years | Yes | 54/293 | 15.6 | **0.03** | 1.4 | 1.0-1.8 | **0.03** | 1.2 | 1.0-1.6 | 0.11 | 22/293  148/2179 | 7.0  6.4 | 0.67 | 1.1 | 0.7-1.7 | 0.67 | 1.0 | 0.7-1.6 | 0.91 |
|  | No | 283/2179 | 11.5 |  |  |  |  |  |  |  |  |  |  |  |  |  |  |  |  |
| 16 years | Yes | 43/245 | 14.9 | 0.11 | 1.3 | 0.9-1.7 | 0.11 | 1.2 | 0.9-1.6 | 0.22 | 25/245 | 9.3 | **0.047** | 1.5 | 1.0-2.3 | **0.045** | 1.4 | 1.0-2.2 | 0.08 |
|  | No | 305/2298 | 11.7 |  |  |  |  |  |  |  | 150/2298 | 6.1 |  |  |  |  |  |  |  |
| Overall | Yes | 149/937 | 13.7 | **<0.01** | 1.3 | 1.1-1.6 | **<0.01** | 1.3 | 1.1-1.6 | **0.01** | 73/937 | 7.2 | 0.17 | 1.2 | 0.9-1.7 | 0.17 | 1.2 | 0.9-1.6 | 0.21 |
|  | No | 155/1351 | 10.3 |  |  |  |  |  |  |  | 84/1351 | 5.9 |  |  |  |  |  |  |  |
| **Food hypersensitivity** | | | | | | | | | | | | | | | | | | | |
| 1-2 years | Yes | 49/363 | 11.9 | 0.99 | 1.0 | 0.8-1.3 | 0.99 | 1.0 | 0.8-1.3 | 0.91 | 19/363  147/2132 | 5.0  6.5 | 0.27 | 0.8 | 0.5-1.2 | 0.27 | 0.8 | 0.5-1.2 | 0.30 |
|  | No | 287/2132 | 11.9 |  |  |  |  |  |  |  |  |  |  |  |  |  |  |  |  |
| 4 years | Yes | 43/258 | 14.3 | 0.22 | 1.2 | 0.9-1.6 | 0.22 | 1.2 | 0.9-1.6 | 0.21 | 20/258 | 7.2 | 0.56 | 1.1 | 0.7-1.8 | 0.56 | 1.1 | 0.7-1.8 | 0.55 |
|  | No | 306/2275 | 11.9 |  |  |  |  |  |  |  | 153/2275 | 6.3 |  |  |  |  |  |  |  |
| 8 years | Yes | 42/323 | 11.5 | 0.86 | 1.0 | 0.7-1.3 | 0.87 | 0.9 | 0.7-1.3 | 0.73 | 19/323 | 5.6 | 0.56 | 0.9 | 0.5-1.4 | 0.56 | 0.9 | 0.5-1.4 | 0.50 |
|  | No | 289/2157 | 11.8 |  |  |  |  |  |  |  | 147/2157 | 6.4 |  |  |  |  |  |  |  |
| 12 years | Yes | 107/582 | 15.5 | **<0.01** | 1.4 | 1.2-1.8 | **<0.01** | 1.4 | 1.1-1.7 | **<0.01** | 57/582 | 8.9 | **<0.01** | 1.6 | 1.2-2.1 | **<0.01** | 1.5 | 1.1-2.1 | **<0.01** |
|  | No | 230/1890 | 10.8 |  |  |  |  |  |  |  | 113/1890 | 5.6 |  |  |  |  |  |  |  |
| 16 years | Yes | 128/616 | 17.2 | **<0.001** | 1.7 | 1.4-2.1 | **<0.001** | 1.6 | 1.3-2.0 | **<0.001** | 69/616 | 10.1 | **<0.001** | 1.9 | 1.4-2.6 | **<0.001** | 1.9 | 1.4-2.5 | **<0.001** |
|  | No | 220/1927 | 10.2 |  |  |  |  |  |  |  | 106/1927 | 5.2 |  |  |  |  |  |  |  |
| Overall | Yes | 159/902 | 15.0 | **<0.001** | 1.6 | 1.3-2.0 | **<0.001** | 1.5 | 1.2-1.9 | **<0.001** | 82/902 | 8.3 | **<0.01** | **1.6** | **1.2-2.2** | **<0.01** | **1.6** | **1.2-2.1** | **<0.01** |
|  | No | 145/1386 | 9.5 |  |  |  |  |  |  |  | 75/1386 | 5.1 |  |  |  |  |  |  |  |
| ^a^ Cases refers to children with any AP-FGID/IBS at 16y. Non-cases refers to children with no AP-FGID at 16y. The column shows the number of cases and non-cases exposed (yes) vs unexposed (no) to asthma/rhinitis/eczema/food hypersensitivity at different ages.  ^b^ Pearson’s chi-squared test. Statistically significant differences of the AR of developing AP-FGID/IBS at 16 years between children exposed and children unexposed to asthma/rhinitis/eczema/food hypersensitivity are shown in bold text.  ^c^ Age-specific and overall (any report from 1y through 16y) associations assessed in a binomial generalized linear model with a log link function. Children with no AP-FGID at 16y were used as the reference group in all analyses. Statistically significant associations are marked in bold text.  *Abbreviations:* AP-FGID, abdominal pain-related functional gastrointestinal disorder; AR, absolute risk; aRR, adjusted relative risk; CI, confidence interval; IBS, irritable bowel syndrome; N, number; RR, relative risk; Y, years. | | | | | | | | | | | | | | | | | | | |
